# Ultrasound-guided repetitive pulsed peripheral magnetic stimulation provides pain relief in refractory glossopharyngeal neuralgia: A case report

**DOI:** 10.1080/24740527.2022.2157250

**Published:** 2023-01-27

**Authors:** James S. Khan, Duncan Westwood, Massieh Moayedi

**Affiliations:** a Wasser Pain Management Centre, Mount Sinai Hospital, Toronto, Ontario, Canada; b Department of Anesthesiology and Pain Medicine, University of Toronto, Toronto, Ontario, Canada; c Department of Dentistry, Mount Sinai Hospital, Toronto, Ontario, Canada; d Centre for Multimodal Sensorimotor and Pain Research, Faculty of Dentistry, University of Toronto, Toronto, Ontario, Canada; e The Study of Pain, University of Toronto Centre, Toronto, Ontario, Canada; f Clinical & Computational Neuroscience, Krembil Research Institute, University Health Network, Toronto, Ontario, Canada

**Keywords:** glossopharyngeal neuralgia, neuropathic pain, magnetic stimulation, neuromodulation, case report

## Abstract

**Aims:**

Repetitive peripheral magnetic stimulation (rPMS) is a novel nonpharmacological treatment modality. This noninvasive approach can stimulate peripheral nerves to provide analgesia through neuromodulation. We report the first case of ultrasound-guided rPMS to treat a case of severe refractory glossopharyngeal neuralgia.

**Methods:**

A 70-year-old female with an 8-year history of glossopharyngeal neuralgia reported refractory pain unresponsive to pharmacological and interventional treatments. After consenting to treatment, the patient received high-frequency rPMS in three different sessions using intermittent theta burst stimulation below motor thresholds. rPMS was applied over the skin directed at the glossopharyngeal nerve identified using ultrasound guidance. Session 1 included 20 min of continuous treatment, session 2 included 40 min of treatment (two 20-min treatments separated by a 10-min break), session 3 included 40 min of treatment (similar to Session 2) repeated daily for 5 days. Pre- and postintervention pain levels were collected with a daily 1-week pain diary and pain questionnaires.

**Results:**

Session 1 led to an immediate 30% decrease in pain after treatment. Session 2 led to a 75% decrease in pain immediately after treatment that remained reduced for approximately 2 days. Session 3 produced complete pain relief immediately after treatment and remained lower for 5 days after treatment and returned to baseline levels at 1 week.

**Conclusion:**

rPMS provided immense but temporary relief in a severe case of refractory glossopharyngeal neuralgia. Further work is needed to determine the most effective regimen to treat complex pain disorders in the head and neck.

## RÉSUMÉ

La stimulation magnétique périphérique répétitive (SPMr) est une nouvelle modalité de traitement non pharmacologique. Cette approche non invasive peut stimuler les nerfs périphériques pour fournir une analgésie par le truchement de la neuromodulation. Nous rapportons le premier cas de SPMR guidée par ultrasons pour traiter un cas de névralgie glossopharyngée réfractaire sévère.**Méthodes**: Une femme de 70 ans avec une histoire de huit ans de névralgie glossopharyngée a fait état d’une douleur réfractaire ne répondant pas aux traitements interventionnels pharmacologiques. Après avoir consenti au traitement, la patiente a reçu une SMPr à haute fréquence au cours de trois séances différentes en utilisant la stimulation thêta-burst intermittente en-deçà des seuils moteurs. La SMPr a été appliquée sur la peau en ciblant le nerf glossopharyngé identifié à l’aide d’un guidage par ultrasons. La séance 1 comprenait 20 minutes de traitement continu, la séance 2 comprenait 40 minutes de traitement (deux traitements de 20 minutes séparés par une pause de 10 minutes), la séance 3 comprenait 40 minutes de traitement (similaire à la séance 2) répétées quotidiennement pendant cinq jours. Les niveaux de douleur pré et post-intervention ont été collectés à l’aide de questionnaires sur la douleur et consignés dans un journal quotidien de la douleur échelonné sur une semaine.**Résultats**: La séance 1 a entraîné une diminution immédiate de 30 % de la douleur après le traitement. La séance 2 a conduit à une diminution de 75 % de la douleur immédiatement après le traitement et cette diminution s’est maintenue pendant environ deux jours. La séance 3 a donné lieu à un soulagement complet de la douleur immédiatement après le traitement; la douleur est ensuite restée plus faible pendant cinq jours après et est revenue aux niveaux de départ au bout d’une semaine.**Conclusion**: La SMPr a procuré un soulagement immense mais temporaire dans un cas grave de névralgie glossopharyngée réfractaire. Des travaux supplémentaires sont nécessaires pour déterminer le régime de traitement le plus efficace pour traiter les troubles douloureux complexes de la tête et du cou.

## Introduction

Neuropathic pain is defined as pain as a result of a lesion or disease of the somatosensory system (within the peripheral or central nervous system),^1^ affecting between 7% and 10% of the general population.^2^ People with neuropathic pain report greater impairments in all aspects of daily living, seek increased attention from health care practitioners, and report more medication use than those with nonneuropathic chronic pain.^3^

Peripheral neuropathic pain is a subset of neuropathic pain and arises from damage or dysfunction of peripheral nerves.^4^ Unfortunately, pharmacological strategies for peripheral neuropathic pain are generally ineffective or associated with significant side effects.^5,6^ Interventional options exist and include peripheral nerve blocks and surgery; however, these techniques are associated with procedural complications such as bleeding, infection, nerve damage, and pain exacerbations.

Neuromodulation is a therapeutic modality where physical energy is directed toward neuronal targets to excite, inhibit, or disrupt normal functioning in a controlled manner.^7^ Electrical neuromodulation has been demonstrated to be effective in the treatment of neuropathic pain; however, typical methods used (i.e., spinal cord stimulators, deep brain stimulation, peripheral nerve stimulators) are limited due to their expense and invasiveness. Though noninvasive neuromodulation devices exist (e.g., transcutaneous electrical stimulation; TENS), these prior applications are limited in their ability to stimulate deep neuronal structures.

Time-varying magnetic fields can be used to electrically activate neuronal targets and can overcome the limitations of surface and implantable electrodes. This method has been growing in popularity with stimulation of cortical structures such as in transcranial magnetic stimulation (TMS). Though TMS has been explored in the management of chronic neuropathic pain with favorable data,^8^ limited investigations have explored magnetic fields directed toward peripheral nerve targets. Nonetheless, these investigations have suggested that repetitive peripheral magnetic stimulation (rPMS) can induce immediate and prolonged analgesia.^9^

Given the need to identify effective and safe treatment options for neuropathic pain, we trialed rPMS on a patient with an 8-year history of refractory glossopharyngeal neuralgia. We combined rPMS with ultrasonography to facilitate anatomic localization and improve magnetic beam placement.

## Materials and Methods

A 70-year-old female with an 8-year history of glossopharyngeal neuralgia resulting from a lingual tumor resection was sent to our clinic for consultation. She had seen several pain physicians at a tertiary academic care center and failed pharmacologic (i.e., neuropathic pain medications, opioids, cannabinoid products) and interventional techniques (nerve blocks, pulsed radiofrequency neuromodulation). Daily mean pain score, collected over the preceding 3 days, was reported to be a 4.2 (SD 2.5) on a 0 to 10 numeric rating scale (NRS). Each day, her pain was low in the morning (mean 1.2, SD 0.8), moderate at midday (average score 5.2, SD 1.8), and severe in the evening (mean 7, SD 1.0).

A trial of rPMS was suggested; prior to proceeding, the patient was informed of rare but potential risks of magnetic stimulation (e.g., local heat, seizures) and possible hemodynamic changes given the anatomic proximity of the glossopharyngeal nerve to the vagus nerve. Institutional ethics approval was waived because the project was a retrospective report of clinically obtained data. The patient provided written consent prior to rPMS treatment that data could be used for publication purposes; the patient did not review the final publication because she died prior to publication.

To identify the approximate location of the glossopharyngeal nerve, the patient’s styloid process was identified on the lateral side of her head using a SonoSite Edge II 5- to 10-MHz linear high-frequency ultrasound probe.^10^ A mark was obtained on the skin overlying the styloid process and this mark was used to position a 70-mm figure-of-eight air film coil directly over the skin mark. rPMS was performed using a Magstim Super Rapid II Stimulator with an intensity set to submotor threshold, identified in a graded fashion starting at 5% of stimulator power output and increased incrementally. The submotor threshold was 21% output of Magstim power.

We conducted three separate sessions of rPMS for this patient using the following stimulation regimen: 50 Hz triplet pulses at 5 Hz with a cycle time of 10s for 120 cycles and a total of 3600 pulses. This regimen was based upon theta burst stimulation for TMS.^11,12^ In the first session, the patient received 20 min of stimulation. The second session occurred 1 week later and included two 20-min stimulation sessions separated by a 10-min break, for a total of 40 min of rPMS. The patient completed a third session 7 weeks later, consisting of two 20-min sessions of rPMS separated by a 10-min break (identical to session 2), repeated daily for 5 days. NRS pain scores and hemodynamics (noninvasive blood pressure measurement and plethysmography) were measured at 5-min increments during stimulation. NRS pain scores were recorded three times a day (8:00 a.m., 12:00 p.m., and 8 p.m.) for 7 days after treatment.

## Statistical Analyses

Descriptive data on the patient’s reported pain scores prior to and after magnetic stimulation are presented, in addition to qualitative reports of pain improvements. No quantitative statistical analyses were performed.

## Results

Prior to treatment, baseline pain was reported as 5/10 on NRS. At the end of the first treatment session, the patient reported an NRS pain score of 3.5/10 (30% immediate pain reduction). One day later, she reported an NRS pain score of 4/10 and 1 week later she reported a score of 4.5/10. Qualitatively, the patient reported that her morning pain was substantially improved, but midday and evening pain NRS remained similar to prestimulation levels.

Baseline pain scores were averaged over the 3 days prior to the second treatment session (4.5/10). After the first 20 min of stimulation, the patient’s NRS pain score was 2/10 (56% immediate pain reduction). At the end of the second 20-min stimulation, NRS pain score was 1/10 (77% reduction from pretreatment pain intensity). The patient reported an average daily NRS pain score of 3.3/10 at both 1 day and 1 week after treatment.

Baseline pain scores were averaged over the 3 days prior to the third treatment session (5.4/10). On 2 of the 5 days of treatment, the patient obtained 100% pain relief (0/10 pain score) immediately after treatment, with an average of pain score of 1.4 over the 5 days. One day after the treatment paradigm (i.e., on day 6), NRS pain scores remained at 2.7/10. At 1 week after the last day of treatment, pain scores returned to a baseline level of 5.3/10. However, as with previous rPMS treatment, morning pain scores showed substantial improvements each day compared to afternoon and evening scores; most notable, the patient reported 0/10 pain on three of the seven mornings, which she had never experienced before. The patient stated that on these mornings, she briefly “got her life back.” The patient self-reported that her level of relief from rPMS was greater than that obtained from any medication or interventional treatment to date.

Throughout treatment sessions, the patient’s vitals (noninvasive blood pressure measurement, heart rate, and pulse oximetry) were monitored. Vital signs were stable throughout all stimulation periods. Subjectively, the patient reported no side effects to stimulation such as local heat, irritation, dizziness, or neurologic issues.

## Discussion

We describe a case of refractory glossopharyngeal neuralgia treated using ultrasound-guided peripheral magnetic stimulation resulting in immediate profound pain reduction after a 40-min session of stimulation. Strengths of this case report include the first documented report of utilizing ultrasound to guide peripheral magnetic stimulation and the use of this therapy in treating a complex neuropathic pain disorder.

Hallgren first proposed stimulating neural tissue with an induced electrical field,^13^ and one of the first applications of this therapy was for peripheral nerve stimulation.^14^ Since then, noninvasive neuromodulation approaches have gained popularity, primarily with stimulation of cortical targets (i.e., TMS).^15^ However, there has been significantly less progress and research on rPMS.

A case series reported five patients with pudendal neuralgia or sciatica treated with 30 to 50 pulses at 75% maximal output of a Magstim 200 device (1.5 Tesla) with the coil directed over the sacral area. This report documented a dramatic immediate effect (90%–100% pain relief) in all patients. Pain relief lasted between 30 min and 56 days.^16^ Another study conducted by Khedr et al. explored PMS in patients with intractable traumatic brachial plexopathy. Thirty-four patients were randomized with a ratio of 2:1 to either the intervention (physical therapy and magnetic stimulation) or control (physical therapy or sham magnetic stimulation).^17^ The intervention group reported 40% pain relief compared to the control arm after 5 sessions and 70% relief after 10 sessions. Pain relief increased to 90% compared to controls after 1 month of therapy. Studies have explored peripheral magnetic stimulation for nonneuropathic pain disorders and demonstrated analgesic benefit for myofascial pain, tendonitis, rotator cuff injury, carpal tunnel syndrome, lumbar spondylosis, acute back pain, and migraines.^18–22^

The case reported here adds to our current understanding of potential applications of rPMS. First, the patient was refractory to conservative, pharmacologic, and advanced pain management therapies (e.g., glossopharyngeal steroid nerve block and pulsed radiofrequency neuromodulation). Targeted rPMS delivered to the glossopharyngeal nerve using ultrasound guidance provided immense and immediate pain relief exceeding the analgesic effect of any previous therapy. After a number of sessions, the patient experienced no pain for several hours, which she had not experienced since the start of her symptoms. Second, our case is the first report of rPMS using image guidance to direct the magnetic beams. Ultrasound has revolutionized pain medicine in its ability to provide real-time accuracy in identifying anatomical structures for interventional procedures (e.g., directing needle placement for nerve blocks, joint injections). Improved accuracy afforded by image guidance has led to superior outcomes such as increased efficacy rates and fewer side effects with pain injection therapy. Similarly, image guidance could allow improved benefit by nerve localization with rPMS.

The mechanism of rPMS analgesia remains unknown. Time-varying magnetic fields convert into electrical energy (Faraday’s law), and positioning magnetic beams over neuronal targets can induce nerve stimulation (i.e., electrical neuromodulation). rPMS is believe to also exert an analgesic effect by interfering with peripheral impulse conduction, activating large afferent peripheral fibers, modulating peripheral nerve and dorsal root ganglion, acting upon endogenous opioidergic pathways, and activating spinal and supraspinal inhibitory mechanisms.^23–25^

Limitations to this case report include limited time intervals between treatment sessions such that prior sessions could influence response to future treatments (i.e., carryover effects). Another limitation that must be considered is the potential for a placebo effect. The patient was aware of the experimental nature of trialing this treatment option, in addition to having serial pain score measurements, so there is reason to believe that a placebo effect could be present. The placebo effect is also enhanced when evaluating pain outcomes due to expectancy-induced analgesia (i.e., expectations for pain relief result in endogenous modulatory mechanisms that result in analgesia).^26^ Furthermore, given that this is a single case, there is a high potential for confounding; for example, the patient was required to remain still for magnetic stimulation, which could induce a relaxed and calm state that could result in improved pain scores rather than a direct effect of rPMS.

Compared to TENS, rPMS may allow for improved comfort, contactless administration, and the ability to target deeper neuronal structures. The latter feature may improve efficacy compared to TENS, because evidence indicates that rPMS provides significantly longer pain relief.^22^ The noninvasive nature of PMS is also advantageous over implantable neuromodulatory devices given that the glossopharyngeal nerve is not an appropriate target for peripheral or spinal cord stimulators. However, additional studies are needed to characterize the effect and mechanisms of PMS, including optimization of stimulation parameters.

## Conclusion

Our case suggests that PMS may produce an immediate yet short-lived analgesic effect for neuropathic pain that has been refractory to prior medical treatment options. Future research should be directed toward identifying the specific treatment regimen (stimulation parameters, dose, and frequency of administrations) required for lasting pain relief or reduction.

## Highlights

Clinical implications:

- Glossopharyngeal neuralgia is an incredibly difficult pain disorder to treat, and use of peripheral magnetic stimulation could be an effective and safe treatment modality.
- Difficult-to-treat oral and facial pain disorders that are a result of a peripheral nerve injury could be treated noninvasively using pulsed magnetic fields.
- The use of ultrasonography in localizing a peripheral nerve for noninvasive neuromodulation is novel and should be further explored.
